# First record of the genus *Dolichosoma* Stephens (Coleoptera, Dasytidae) from China, with two newly-recorded species

**DOI:** 10.3897/BDJ.12.e129639

**Published:** 2024-07-09

**Authors:** Jialin Miao, Haoyu Liu, Junbo Tong, Yuxia Yang

**Affiliations:** 1 Key Laboratory of Zoological Systematics and Application, School of Life Sciences, Hebei University, Baoding, China Key Laboratory of Zoological Systematics and Application, School of Life Sciences, Hebei University Baoding China; 2 Hebei Basic Science Center for Biotic Interaction, Hebei University, Baoding, China Hebei Basic Science Center for Biotic Interaction, Hebei University Baoding China

**Keywords:** new faunistic record, alpha taxonomy, China, Cleroidea, Dasytidae

## Abstract

**Background:**

*Dolichosoma* Stephens, 1830 is small genus belonging to the tribe Dasytini of the family Dasytidae (Coleoptera, Cleroidea), with two subgenera and five species hitherto known. It is widespread in the Palearctic Region of Eurasia, but has never been reported from China until now.

**New information:**

The genus *Dolichosoma* Stephens, 1830 is reported from China for the first time, with discoveries of two newly-recorded species, including D. (Dolichomorphus) femorale Morawitz, 1861 and D. (Dolichosoma) lineare (Rossi, 1794) from Xinjiang Autonomous Region. They are re-described in detail and illustrated with habitus, ultimate abdominal tergites and sternites and genitalia of both sexes, as well as tarsal claws of male. In addition, a macrohabitat photograph and a distribution map of the two species occurring in China are provided.

## Introduction

The genus *Dolichosoma* was proposed by Stephens (1830), with *Lagrialinearis* Rossi, 1794 as the type species. It is currently classified in the tribe Dasytini of Dasytidae (Mayor 2007, Gimmel and Mayor 2019). The adults of *Dolichosoma* can be easily distinguished from other dasytid beetles by its extremely slender body. This genus has been divided into two subgenera, D. (Dolichosoma) Stephens, 1830 and D. (Dolichomorphus) Fiori, 1905 (Constantin 2007, Gimmel and Mayor 2019, Liberti 2021), which are different as follows. In D. (Dolichomorphus), the pronotum is nearly as long as wide or transverse; the elytra are densely covered with whitish pubescence and scattered with a few erected blackish setae on surface; and the ultimate maxillary palpomere is large (about 4.0 times longer and 1.5 times wider than the penultimate palpomere) and securiform in shape. In contrast to D. (Dolichosoma), where the pronotum is significantly longer than wide; the elytra are sparsely covered with recumbent scale-like whitish pubescence on surface; and the ultimate maxillary palpomere is of normal size (about 3.0 times longer and 1.1 times wider than the penultimate palpomere) and cylindrical in shape. At present, there are one and three species included in the two subgenera, respectively (Liberti 2021). Additionally, *Dolichosoma* is considered to be endemic to the Palearctic Region (Gimmel and Mayor 2019), but *D.indicum* Pic, 1923 is located in India, which remains poorly known and is placed in *incertae sedis* at the moment (Liberti 2021).

*Dolichosoma* is widespread in the Palearctic Region of Eurasia and has been reported in the neighbourhood of China. In the present study, we assembled some material from Xinjiang Autonomous Region of China and identified it as D. (Dolichomorphus) femorale Morawitz, 1861 and D. (Dolichosoma) lineare (Rossi, 1794), which represent the first records of *Dolichosoma* in the Chinese fauna. To make them better known, we will re-describe and illustrate the two species in detail, also will provide illustrations of their female genitalia for the first time.

## Materials and methods

In this study, we will adhere to the conventional taxonomic classification of dasytid beetles as a separate family, Dasytidae (Majer 1994, Majer 2002, Mayor 2007, Bocakova et al. 2012, Constantin 2021), rather than considering them as a subfamily within Melyridae (Gimmel and Mayor 2019). The specimens examined in this study are deposited in the Institute of Zoology, Chinese Academy of Sciences, Beijing, China (IZAS) and the Museum of Hebei University, Baoding, China (MHBU).

The specimens were initially soaked in water to achieve softening, followed by the separation of their abdomen. The separated abdomen was then immersed in a 10% sodium hydroxide (NaOH) solution and subjected to heating at a constant temperature for several minutes using a metal bath. Once the fat had dissolved, it was transferred to a Nikon SMZ1500 stereomicroscope for dissection of the pygidium, ultimate abdominal ventrite and genitalia. To facilitate observation, the spiculum gastrale, tegmen and median lobe were individually isolated. The ovipositor was stained with haematoxylin. Subsequently, the dissected genitalia were placed on a glass slide with glycerol and photographed using a Leica M205A stereomicroscope before being stored in glycerol for preservation purposes. A Canon EOS 70D digital camera was employed to capture images of habitus which were later processed using Helicon Focus 7 software. Adobe Photoshop CC 2019 version 20.0.4 was utilised for editing purposes regarding plate preparation. The body length was measured from the anterior margin of the head to the elytral apices and width at the humeri. Terminology of genital segments follows Lawrence et al. (2010) and that of genitalia follows Gimmel and Mayor (2019).

The distribution map was prepared by ArcMap 10.8 and edited in Photoshop CC 2019 20.0.4, based on the distribution information of the present studied material.

## Taxon treatments

### Dolichosoma

Stephens, 1830

410B3288-D0C8-51EC-8308-2B2B2E979BE2

Dolichosoma (Dolichosoma) Stephens, 1830 - Stephens 1830: 318.
Dolichosoma (Dolichomorphus) Fiori, 1905 - Fiori 1905: 81.
Dolichosoma (Dolichosoma) lineare (Rossi, 1794)

#### Diagnosis

Body extremely slender, blackish-green, covered with whitish pubescence on elytra and a few erected black setae on head and elytral apices (sometimes also on pronotum). Ultimate abdominal ventrite slightly emarginate in middle of posterior edge, present with short central process at anterior edge (Fig. 3A and Fig. 6A). Aedeagus: median lobe with apical limb much longer than basal limb, which are vertical to each other; internal sac very short and covered with two lines of large and highly sclerotised black spines (Fig. 3E and Fig. 6E); tegmen with lateral processes parallel, present with two lobes at apex (Fig. 3C and Fig. 6C).

#### Distribution

Palearctic Region of Eurasia.

### Dolichosoma (Dolichomorphus) femorale

Morawitz, 1861

38E7615B-F770-50E7-8FBC-DCE79DD15634

Dolichosoma femorale Morawitz, 1861 - Morawitz 1861: 317 (type locality: Russia); Kiesenwetter 1863: 644; Kiesenwetter 1867b: 137, 139; Schilsky 1894a: 235; Schilsky 1894b: n. 45; Schilsky 1897: n. 34 X; Fiori 1908: 240; Porta 1929: 123; Pic 1937: 108; Kaszab 1955: 117; Kaszab 1977: 59; Majer 1986a: 127; Majer 1986b: 313; Lohse 1992: 22; Liberti 1995: 21; Majer 2005: 157.
Psilothrix (subg. Dolichomorphus) rufimanus Fiori, 1905 - Fiori 1905: 81; Pic 1937: 108.

#### Materials

- **Type status:** Other material. **Occurrence:** recordedBy: Shuyong Wang; individualCount: 2; sex: 2 males; lifeStage: adult; occurrenceID: 273F628A-99C9-5F6A-AA49-FA3AD34115BB; **Location:** country: China; stateProvince: Xinjiang; county: Qinghe; locality: Ertai; verbatimElevation: 940 m; **Event:** year: 1960; month: 7; day: 2; **Record Level:** institutionID: Institute of Zoology, Chinese Academy of Sciences; institutionCode: IZAS
- **Type status:** Other material. **Occurrence:** recordedBy: Chunpei Hong; individualCount: 1; sex: 1 male; lifeStage: adult; occurrenceID: 7FC79093-0AD9-54A3-B7E2-1FEBBF9AC5A9; **Location:** country: China; stateProvince: Xinjiang; county: Manas; locality: Shihezi; verbatimElevation: 415–550 m; **Event:** year: 1957; month: 6; day: 7; **Record Level:** institutionID: Institute of Zoology, Chinese Academy of Sciences; institutionCode: IZAS
- **Type status:** Other material. **Occurrence:** recordedBy: Guang Wang; individualCount: 1; sex: 1 female; lifeStage: adult; occurrenceID: 702EE07E-F4D9-5539-AC0F-DD73E85FEE24; **Location:** country: China; stateProvince: Xinjiang; county: Manas; locality: Shihezi; verbatimElevation: 460–510 m; **Event:** year: 1957; month: 6; day: 7; **Record Level:** institutionID: Institute of Zoology, Chinese Academy of Sciences; institutionCode: IZAS
- **Type status:** Other material. **Occurrence:** recordedBy: Chunpei Hong; individualCount: 1; sex: 1 female; lifeStage: adult; occurrenceID: E76D42B8-3215-5563-B2F0-9B0305815DB9; **Location:** country: China; stateProvince: Xinjiang; county: Manas; verbatimElevation: 385 m; **Event:** year: 1957; month: 6; day: 9; **Record Level:** institutionID: Institute of Zoology, Chinese Academy of Sciences; institutionCode: IZAS
- **Type status:** Other material. **Occurrence:** recordedBy: Shijun Ma, Kailing Xia, Yonglin Chen; individualCount: 1; sex: 1 female; lifeStage: adult; occurrenceID: 08FE7711-C5DF-58DA-BF1B-0EA590223610; **Location:** country: China; stateProvince: Xinjiang; county: Barkol; **Event:** year: 1955; month: 6; day: 28; **Record Level:** institutionID: Institute of Zoology, Chinese Academy of Sciences; institutionCode: IZAS

#### Description

**Figure 1.**
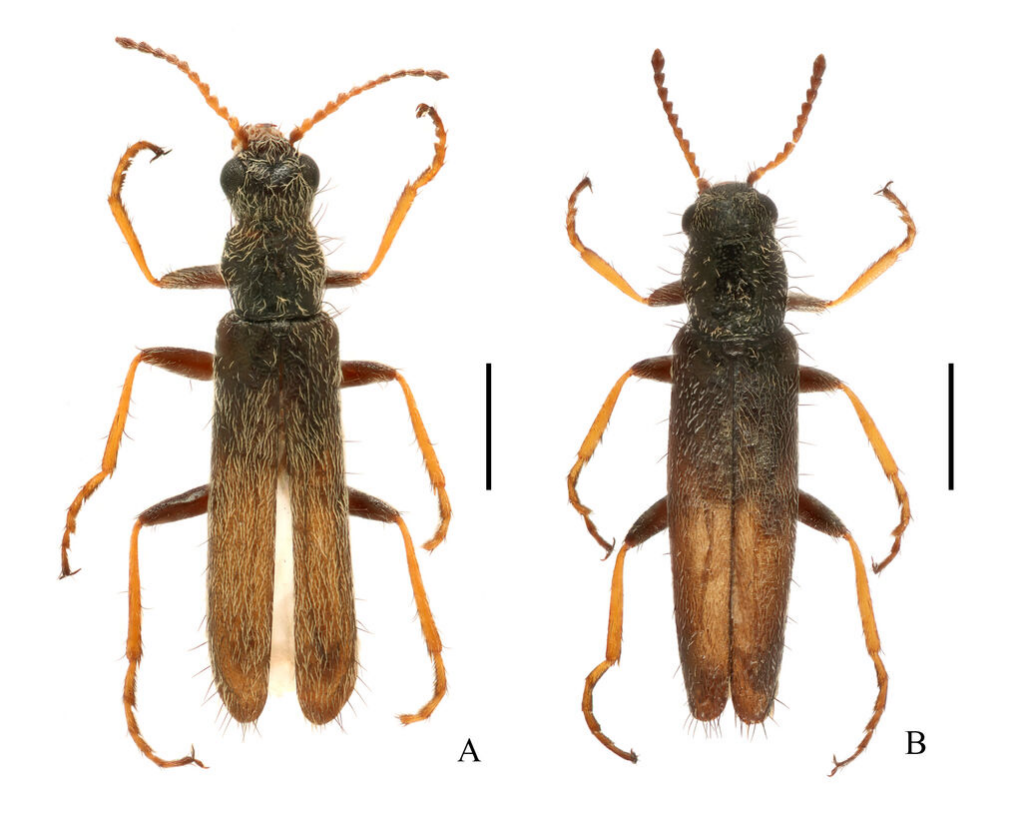
Habitus of Dolichosoma (Dolichomorphus) femorale Morawitz, 1861, dorsal view: **A** male; **B** female. Scale bars: 1.0 mm.

**Male** (Fig. 1A). Body slender, length 3.7–4.7 mm, width 0.8–1.0 mm.

Body green with strong metallic lustre. Antennae yellow, antennomeres 1 and 8–11 more or less darkened. Tibiae and tarsi yellow, tarsomeres more or less darkened. Body densely and coarsely punctate on surface, densely covered with rather short and recumbent whitish pubescence and scattered with a few erected black setae on head, pronotum and elytra.

Head width across eyes as wide as pronotum. Antennae serrate and quite short, extending to posterior margin of pronotum when inclined, antennomeres 2–10 triangular and nearly as long as wide, subequal in length, 11 fusiform and pointed at apices. Ultimate palpomere large and securiform, about 4.0 times longer and 1.5 times wider than the penultimate palpomere, with inner edge obviously angled near base.

Pronotum as long as wide, widest near middle, with anterior margin feebly arcuate, lateral margins moderately arcuate, posterior margin nearly straight, anterior and posterior angles widely rounded.

Elytra parallel-sided, round at apices, 3.2–3.3 times longer than wide at humeri, 3.8–4.0 times longer than pronotum.

**Figure 2.**
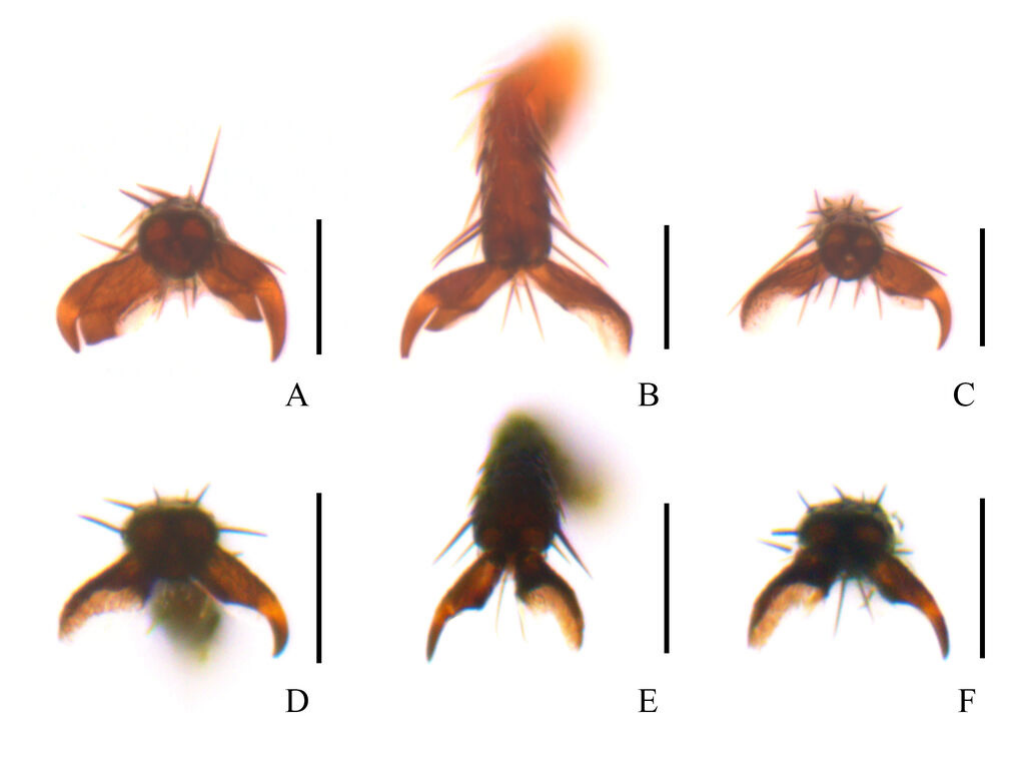
Male tarsal claws of *Dolichosoma* species, frontal views: **A**, **B**, **C** D. (Dolichomorphus) femorale Morawitz, 1861; **D**, **E**, **F** D. (Dolichosoma) lineare (Rossi, 1794). **A**, **D** protarsal claws; **B**, **E** mesotarsal claws; **C**, **F** metatarsal claws. Scale bars: 0.1 mm.

Tarsal claws (Fig. 2A–C) asymmetrical in the structure, each protarsal outer claw provided with a well-developed and large basal appendicle, while moderately large on inner claw; mesotarsal outer claw with a moderately large basal appendicle, while reduced on inner claw; metatarsal outer claw without basal appendicle, while weakly developed on inner claw.

**Figure 3.**
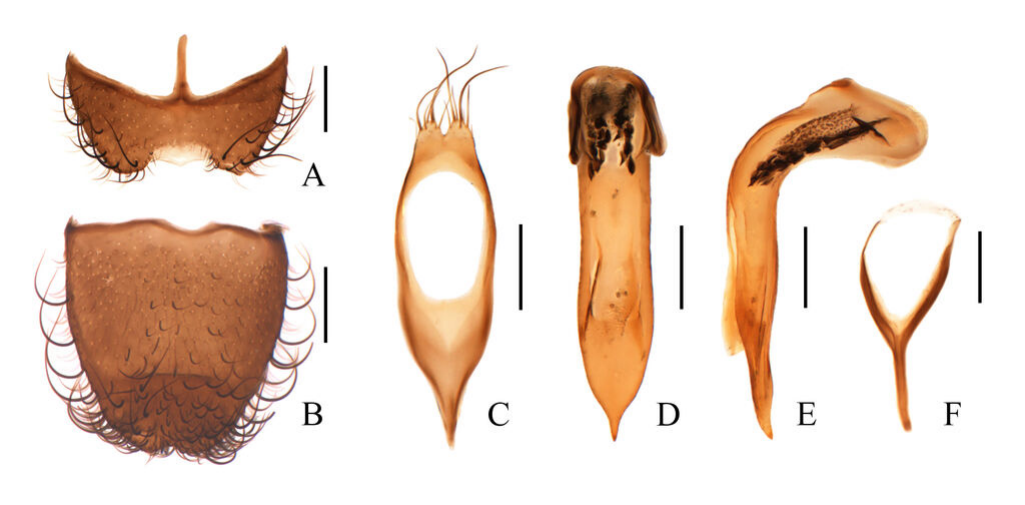
Dolichosoma (Dolichomorphus) femorale Morawitz, 1861, male: **A** ultimate abdominal ventrite (apical sternite), ventral view; **B** pygidium (apical tergite), dorsal view; **C** tegmen, ventral view; **D** median lobe, ventral view, **E** median lobe, lateral view; **F** spiculum gastrale, ventral view. Scale bars: 0.2 mm.

Ultimate abdominal ventrite (Fig. 3A) 1.4–1.6 times as wide as long, largely and trapezoidally emarginate in middle of posterior margin, membranous behind the middle emargination, rounded at apices of posterior-lateral angles, present with short central process at anterior margin, surface covered with long black setae along lateral margins. Pygidium (Fig. 3B) 1.0–1.2 times longer than wide, feebly narrowed posteriorly, hardly emarginate in middle of posterior margin, shallowly and roundly emarginate in middle of anterior margin, with antero-lateral angles feebly protruding, surface covered with short black setae in centre and very long black setae along lateral margins. Aedeagus: tegmen (Fig. 3C) nearly elliptic, strongly narrowed basally and pointed at base, parallel-sided, shortly bilobed and lateral lobes covered with a few long setae; median lobe (Fig. 3D) strongly bent dorsally, with apical limb at an angle of 90˚ with basal limb, apical limb clearly longer than basal limb, strongly narrowed apically and pointed at apex; internal sac (Fig. 3C–D) short, fitted with two lines of large black spines. Spiculum gastrale (Fig. 3F) Y-shaped.

**Figure 4.**
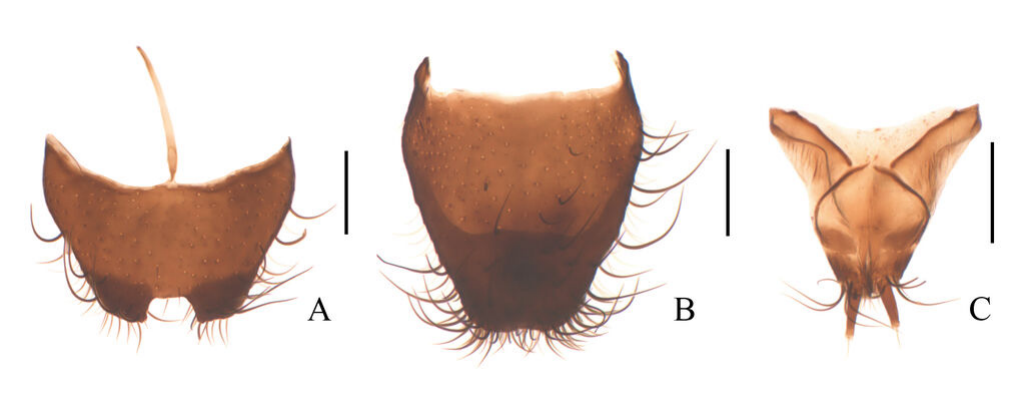
Dolichosoma (Dolichomorphus) femorale Morawitz, 1861, female: **A** ultimate abdominal ventrite (apical sternite), ventral view; **B** pygidium (apical tergite), dorsal view; **C** ovipositor, ventral view. Scale bars: 0.2 mm.

**Female** (Fig. 1B). Similar to male, but body larger, 4.4–4.7 mm in length, width 1.0–1.1 mm; head width across eyes narrower than pronotum, eyes smaller, antennae slightly shorter, ultimate maxillary palpomere with inner edge widely rounded. Pronotum feebly wider than long. Ultimate abdominal ventrite (Fig. 4A) longer, strongly narrowed posteriorly, present with longer central process at anterior margin. Pygidium (Fig. 4B) strongly narrowed posteriorly, nearly straight at anterior margin, with antero-lateral angles obviously protruding. Ovipositor (Fig. 4C) stout and membranous, gonostylus feebly long and nearly cylindrical, transverse coxital baculus long and arched, baculus oblique and nearly as long as transverse coxital baculus.

#### Diagnosis

This species is the sole member of the subgenus Dolichomorphus; it can be distinguished from all other species of *Dolichosoma* by the pronotum nearly as long as wide or transverse; elytra densely covered with short and recumbent whitish pubescence and scattered with a few erected blackish setae on surface; and the ultimate maxillary palpomere extremely large (about 4.0 times longer and 1.5 times wider than the penultimate palpomere) and securiform; pro- and mesotarsal claws (Fig. 2A–C) provided with moderately or well-developed basal appendicles.

#### Distribution

China (Xinjiang, Fig. 8), Italy, Croatia, Greece, Bulgaria, Ukraine, Russia (throughout the temperate zone of country from Saint Petersburg to Vladivostok), Kyrgyzstan, Uzbekistan, Kazakhstan, Mongolia, Hungary and other Palearctic countries.

### Dolichosoma (Dolichosoma) lineare

(Rossi, 1794)

D694190C-68DB-5B79-B047-AF05250E5405

Lagria linearis Rossi, 1794 - Rossi 1794: 92 (type locality: Italy); Stephens 1830: 320; Redtenbacher 1858: 547; Kiesenwetter 1863: 642; Thomson 1864: 146; Kiesenwetter 1867a: 119; Kiesenwetter 1867b: 137; Mulsant and Rey 1868: 269; Seidlitz 1891a: 489; Seidlitz 1891b: 522; Schilsky 1897: nr. 26; Reitter 1911: 289; Pic 1918: 2; Pic 1924: 57, 83; Porta 1929: 123; Pic 1937: 106; Horion 1953: 140; Kaszab 1955: 118; Allenspach and Wittmer 1979: 110; Lohse 1979: 89; Majer 1990: 97; Constantin and Klausnitzer 1996: 196; Sparacio 1997: 106; Bahillo de la and Lopez-Colon 2002: 145; Liberti and Focarile 2005: 29, 35; Mayor 2007: 407.
Tillus filiformis Panzer, 1799 - Panzer 1799: 17; Illiger 1801: 84.
Dolichosoma filum Fairmaire, 1860 - Fairmaire 1860: 630; Constantin 2007: 167.
Dolichosoma subdensatum Mulsant and Rey, 1868 - Mulsant and Rey 1868: 273; Schilsky 1897: nr. 26.
Dolichosoma submicaceum Mulsant and Rey, 1868 - Mulsant and Rey 1868: 274; Schilsky 1897: nr. 26.
Dolichosoma subnodosum Mulsant and Rey, 1868 - Mulsant and Rey 1868: 274; Schilsky 1897: nr. 26.
Dolichosoma tenuiforme Horn, 1880 - Horn 1880: 150–151.

#### Materials

- **Type status:** Other material. **Occurrence:** recordedBy: Zhengzhong Huang; individualCount: 5; sex: 3 males, 2females; lifeStage: adult; occurrenceID: E8F84560-5E03-5FD3-A8C4-19349CEBFAEF; **Location:** country: China; stateProvince: Xinjiang; county: Burjin; locality: Hemu; verbatimElevation: 1333 m; **Event:** year: 2023; month: 6; day: 16; **Record Level:** institutionID: Museum of Hebei University; institutionCode: MHBU

#### Description

**Figure 5.**
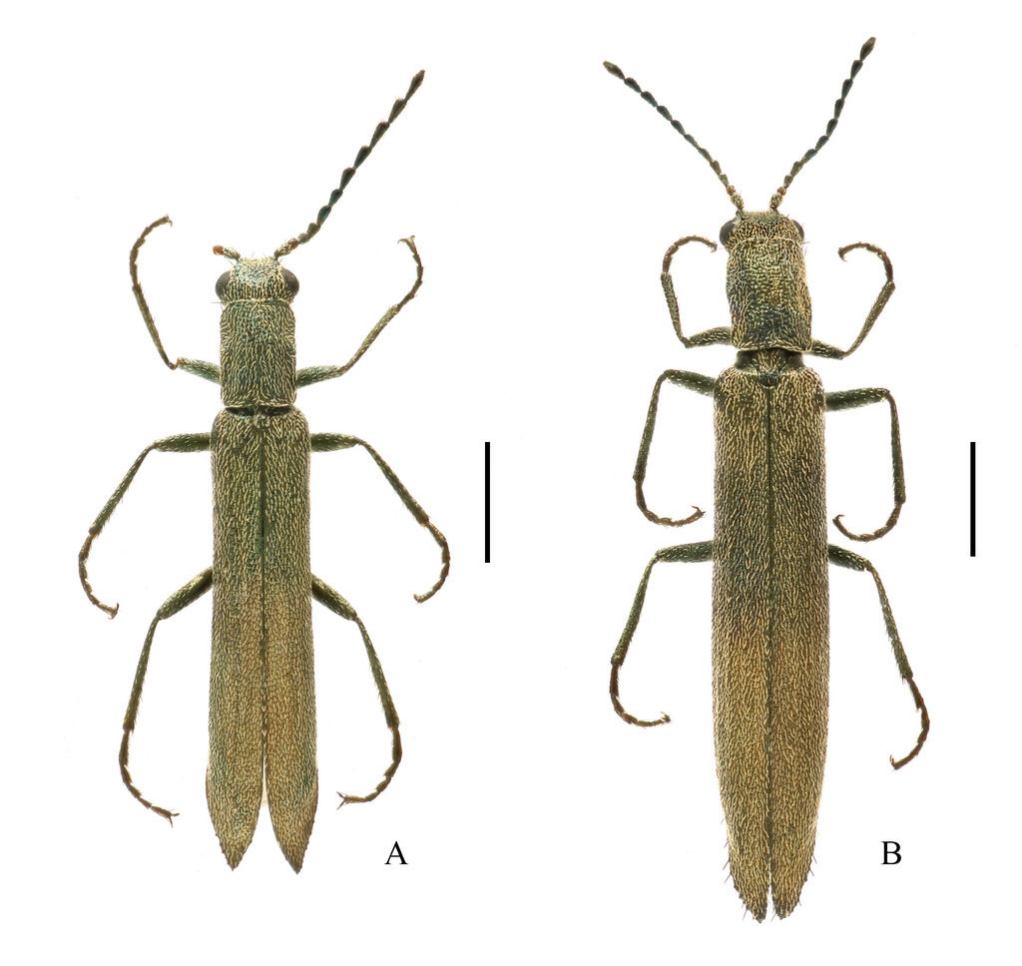
Habitus of Dolichosoma (Dolichosoma) lineare (Rossi, 1794), dorsal view: **A** male; **B** female. Scale bars: 1.0 mm.

**Male** (Fig. 5A). Body extremely slender, length 4.4–5.2 mm, width 0.7–0.8 mm.

Body green with strong metallic lustre. Antennae black, antennomeres 1 green with metallic lustre, 2 yellowish-brown, 2–11 without metallic lustre. Body finely and shallowly punctate on surface, sparsely covered nearly scale-like, short and recumbent whitish pubescence and scattered with only a few erected black setae on head and elytral apex (usually not on pronotum).

Head width across eyes 1.1–1.2 times as wide as pronotum. Antennae serrate and slightly longer, extending to basal 1/5 length of elytra when inclined, antennomere 2 globular, 3–10 triangular, nearly 1.0–3.0 times as long as wide, progressively increased in length, 10 longest and ca. 2.5–2.7 times longer than 3, 11 fusiform and pointed at apices. Ultimate palpomere normal in size and approximately cylindrical, about 3.0 times longer and 1.1 times wider than the penultimate palpomere.

Pronotum parallel-sided, 1.3–1.5 times as long as wide, with anterior margins nearly straight, posterior margin feebly bisinuate, anterior and posterior angles widely rounded.

Elytra parallel-sided, tapered at apices, 4.6–4.9 times longer than wide at humeri, 5.9–6.2 times longer than pronotum.

Tarsal claws (Fig. 2D–F) slightly asymmetrical in the structure, each protarsal inner claw without basal appendicle, while weakly developed on outer claw, meso- and metatarsal outer claws without basal appendicles, while weakly developed on inner claws.

**Figure 6.**
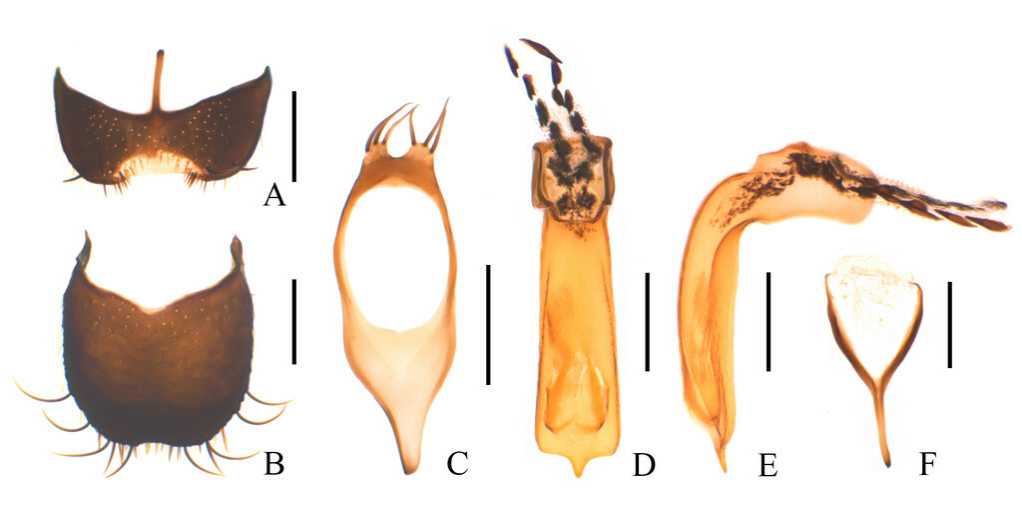
Dolichosoma (Dolichosoma) lineare (Rossi, 1794), male: **A** ultimate abdominal ventrite (apical sternite), ventral view; **B** pygidium (apical tergite), dorsal view; **C** tegmen, ventral view; **D** median lobe, ventral view; **E** median lobe, lateral view; **F** spiculum gastrale, ventral view. Scale bars: 0.2 mm.

Ultimate abdominal ventrite (Fig. 6A) 1.6–1.7 times as wide as long, largely and roundly emarginate in middle of posterior margin, membranous behind the middle emargination, rounded at apices of posterior-lateral angles, present with short central process at anterior margin, surface covered with short black setae along lateral margins. Pygidium (Fig. 6B) 1.1–1.3 times longer than wide, feebly narrowed posteriorly, hardly emarginate in middle of posterior margin, largely and triangularly emarginate in middle of anterior margin, with antero-lateral angles obviously protruding, surface covered with a few long black setae along lateral margins. Aedeagus: tegmen (Fig. 6C) nearly elliptic, strongly narrowed basally and round at base, parallel-sided, shortly bilobed and lateral lobes covered with a few short setae; median lobe (Fig. 6D) strongly bent dorsally, with apical limb at an angle of 90˚ with basal limb, apical limb clearly longer than basal limb, feebly widened apically, then abruptly narrowed and pointed at apex; internal sac (Fig. 6D–E) short, fitted with two lines of large black spines at the apical part and several tiny spines at the base. Spiculum gastrale (Fig. 6F) Y-shaped.

**Figure 7.**
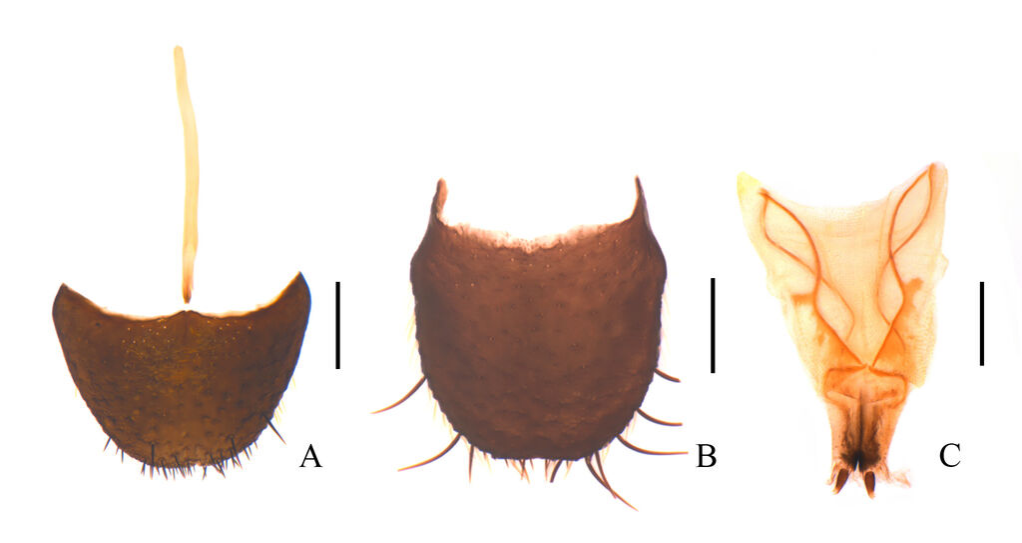
Dolichosoma (Dolichosoma) lineare (Rossi, 1794), female: **A** ultimate abdominal ventrite (apical sternite), ventral view; **B** pygidium (apical tergite), dorsal view; **C** ovipositor, ventral view. Scale bars: 0.2 mm.

**Female** (Fig. 5B). Similar to male, but body larger, 5.8–6.4 mm in length, width 0.9–1.0 mm; antennae slightly shorter, extending to posterior margin of pronotum when inclined. Elytra much longer, about 5.1–5.2 times as long as wide, 6.7–6.8 times longer than pronotum. Ultimate abdominal ventrite (Fig. 7A) longer, strongly narrowed posteriorly, hardly emarginate in middle of posterior margin, present with a longer central process at anterior margin. Pygidium (Fig. 7B) feebly narrowed posteriorly, hardly emarginate in middle of anterior margin, with antero-lateral angles obviously protruding. Ovipositor (Fig. 7C) stout and membranous, gonostylus short and conical, transverse coxital baculus short and oblique, baculus helical and much longer than transverse coxital baculus.

#### Diagnosis

This species is very similar to D. (Dolichosoma) simile (Brullé, 1832) and can be distinguished by the combination of following characters: pronotum not covered with erected black setae on surface; elytra tapered at apices, present with 2–3 weakly developed, but visible longitudinal costae. Unlike in *D.simile*, the pronotum is covered with erected black setae on the surface; elytra are rounded at apices, absent with visible longitudinal costae (Liberti 2009).

#### Distribution

**Figure 8.**
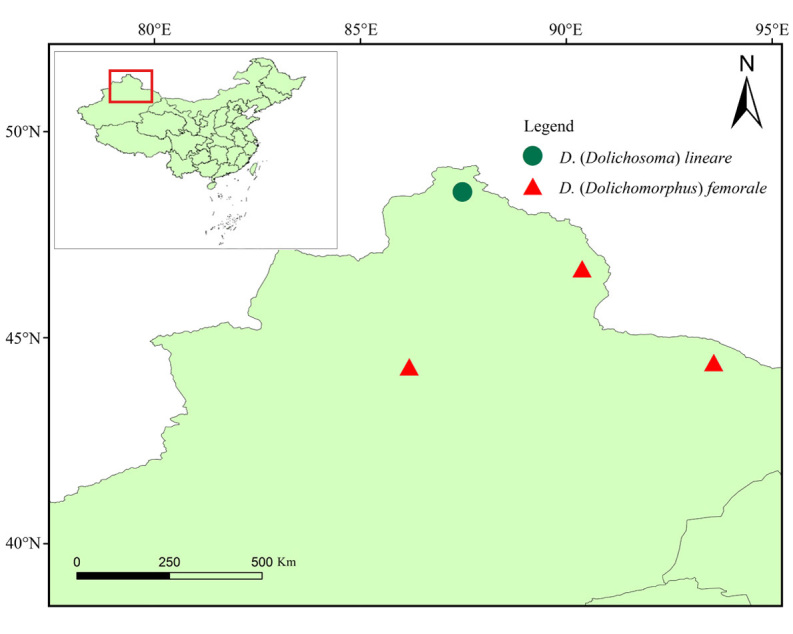
Distribution map of *Dolichosoma* species in China.

**Figure 9.**
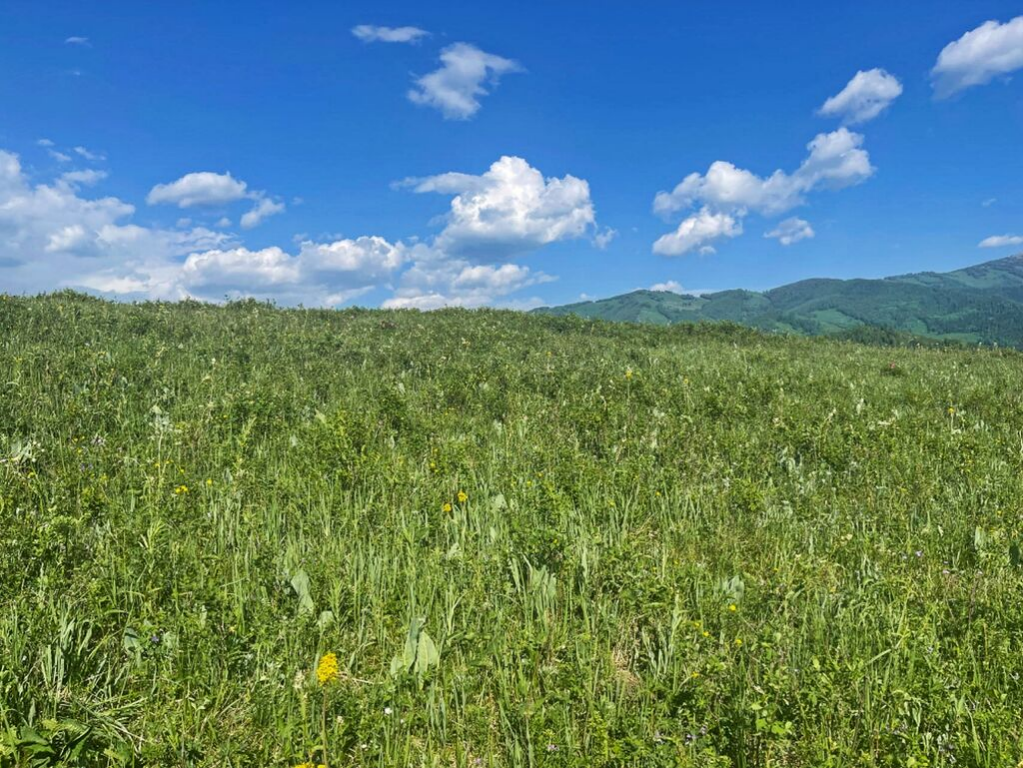
Macrohabitat of Dolichosoma (Dolichosoma) lineare (Rossi, 1794) in Hemu Township, Burjin County, Xinjiang Autonomous Region of China. Photographed by Dr. Zhengzhong Huang in June 2023.

China (Xinjiang, Figs 8, 9), Andorra, Austria, Belgium, Bahrain, Belarus, Croatia, Russia (throughout the temperate zone of country from Saint Petersburg to Vladivostok), Czech Repubic, Denmark, Estonia, Great Britain, Finland, France, Germany, Hungary, Italy, Latvia, Lithuania, Netherlands, Norway, Poland, Romania, Slovakia, Spain, Sweden, Switzerland, Ukraine, East Siberia and Kazakhstan.

## Supplementary Material

XML Treatment for Dolichosoma

XML Treatment for Dolichosoma (Dolichomorphus) femorale

XML Treatment for Dolichosoma (Dolichosoma) lineare
